# *BRCA1* methylation in newborns: genetic disposition, maternal transfer, environmental influence, or by chance only?

**DOI:** 10.1186/s13148-018-0566-0

**Published:** 2018-10-22

**Authors:** Per Eystein Lønning, Stian Knappskog

**Affiliations:** 1 Department of Clinical Science, University of Bergen, Bergen, Norway; 2 Department of Clinical Oncology, Haukeland University Hospital, Bergen, Norway

**Keywords:** *BRCA1*, Hypermethylation, Constitutive methylation, Ovarian cancer, Breast cancer, White blood cells

## Abstract

In this letter, we respond to and discuss the recent publication by Al-Moghrabi et al.: *Methylation of BRCA1 and MGMT genes in white blood cells are transmitted from mothers to daughters.* We discuss their findings with emphasis on two other recently published papers and argue that their data allows no conclusion regarding the transmission of *BRCA1* methylation from parent to child.

The recent publication by Al-Moghrabi et al. [1] reporting *BRCA1* methylation in a significant number of newborn girls reveals interesting data. Many of their findings are consistent with recent data reported by our group [2].

Analyzing 295 newborns, Al-Moghrabi and colleagues found WBC *BRCA1* promoter methylation in 9.9%. Similarly, among *n* = 611 newborns, we found *BRCA1* methylation in 7.0%. Among healthy controls aged 15–50 years, they found *BRCA1* methylation among 25 out of 268 women (9.3%), somewhat contrasting our finding of methylation among 153 out of 3602 (4.2%) of adult healthy women, with a slight reduction during aging. This contrast may have methodological explanations. Considering the methods applied in the two studies, they differ somewhat in respect to which CpGs that were included in the different assays. While the same primers are used in Al-Moghrabi and colleagues’ MSP-assay, our qPCR assay includes a probe covering three additional CpGs. Although qPCR is typically more sensitive than MSP, it may be that the higher number of CpGs covered results in a more stringent threshold for positive reactions. This is depicted in Fig. 1. Notably, the two studies were also conducted in different parts of the world, and potential differences could be related to environmental influence, including diet [3] as well as ethic differences. Thus, there are examples of biologically functional SNPs, like the *MDM2* SNP285G/C variant, affecting cancer risk [4], that is limited to certain ethnic groups [5].

**Fig. 1:**
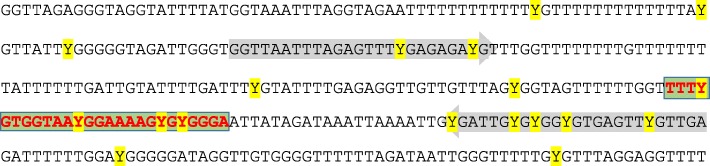
Region of the *BRCA1* promoter (bisulfite converted) covered by the MSP assay performed by Al-Moghrabi et al. [1] and the qPCR assay performed by Lønning et al. [2]. CpG dinucleotides (potential methylation sites) are highlighted in yellow and “Y” indicates that the C in CpG could be either C or T after bisulfite conversion. Primers used in both assays are highlighted by gray background. The additional probe used by Lønning et al. is highlighted by green background and red font

Notably, analyzing samples by pyrosequencing, we found *BRCA1* methylation to be mosaic, in most cases affecting less than 10% of the *BRCA1* alleles. However, comparing methylation frequency among newborns to adults (control population of *n* = 3602 with the addition of a separate group of 292 young females reported in our study), the difference in methylation frequency between newborns and adults was highly significant (Fisher’s exact test; *p* = 0.0021). While we had no access to repeated sampling over time in individuals, this finding is consistent with constitutional methylation but with a slight loss during lifetime. Thus, constitutional methylation patterns are known to change with age [6–8].

What are the potential implications of these findings? While several small studies have indicated normal tissue *BRCA1* methylation to be associated with an elevated risk of breast cancer in general [9, 10] and early breast cancer [11], a final conclusion warrants evaluation in larger cohorts. As for ovarian cancer, analyzing two independent cohorts of 934 ovarian cancer patients versus 1698 controls and 607 patients versus 1984 controls [2], we found WBC *BRCA1* methylation to be associated with an OR of 2.91 (95% CI 1.85–4.56) and 2.22 (95% CI 1.40–3.52), respectively, for high-grade serous ovarian cancer. In contrast, no elevated risk for either low-grade serous ovarian cancer or ovarian cancers harboring other histologic types was detected. These findings further emphasize the potential biological and clinical implications of the results from Al-Moghrabi and colleagues.

The findings, however, raise a number of questions remaining to be addressed. While in our study, *BRCA1* methylation revealed a trend to become less frequent with age across all age groups; the major difference between newborns and adults seemed to occur during the first two decades of life. Thus, while *BRCA1* methylation has been found associated with cancer risk, notably, the ORs have been calculated based on age-matched controls; thus, methylation at birth may not automatically be inferred as a risk factor. We lack definite evidence confirming that individuals carrying *BRCA1* methylation at age 60 carried the same methylation status at birth, although it may be reasonable to assume so. Most cancers likely develop for years before becoming clinically detectable; thus, the finding of an elevated OR for cancer associated with *BRCA1* methylation suggests this methylation has been present for a substantial time period.

The final question relates to the potential cause of constitutional *BRCA1* methylation. In a recent paper, Evans et al. [12] reported a 5′UTR variant in the *BRCA1* promoter to be associated with methylation. Analyzing 49 families with a high incidence of breast and/or ovarian cancer, WBC promoter methylation was detected in two index patients. In these two families, constitutive *BRCA1* methylation was associated with the 5′UTR variant. The *BRCA1* methylation was detected in tissue across all three germ layers. Importantly, in these individuals, methylation was detected in 100% of the 5′UTR-variant-containing *BRCA1* alleles. While our finding of *BRCA1* methylation among healthy carriers [2] also could be associated with genetic variants, several lines of indications argue against such an explanation. Firstly, we found a low incidence of methylated alleles (< 10%), indicating mosaicism. Secondly, the incidence of affected individuals in the population was much higher (4%). Third, the OR for high-grade serous ovarian cancer of 2–3, contrasting the high-risk families reported by Evans et al. Finally and importantly, we found *BRCA1* methylation in both ancient *BRCA1* haplotypes. While these findings argue against influence from a single *cis*-acting factor, it does not exclude the possibility that certain SNP variants may be associated with an elevated risk of *BRCA1* promoter methylation. Further studies are warranted to clarify this issue.

In the title of their manuscript, Al-Moghrabi et al. advocate a mother to daughter transmission of *BRCA1* methylation. Among 20 *BRCA1* methylated mothers, in 290 mother-daughter pairs, they found WBC methylation in 4 of their daughters (20%). From the figures presented in their Table 1, it is not possible to extract how many of the daughters in the 290 mother-daughter pairs that harbored methylation, but 30 out of a total of 302 newborns were found methylated. Assuming these 30 to be among the 290 daughters in mother-daughter pairs, the number of methylated daughters without methylated mother will be 26 out of 270 (9.6%). Testing the hypothesis that *BRCA1* methylation is transmissible from mother to daughter, based on these data, yields a *p* value > 0.10 (Fisher’s exact test). In the extreme and unlikely case that a maximum number of methylated newborns (*n* = 12) are not among the 290 mother-daughter pairs, one would still only reach a *p* value of 0.03. While their hypothesis suggesting an association between *BRCA1* methylation status in mothers and daughters is interesting, we are concerned that the number of observations reported does not allow any statement in this respect for the moment. But we encourage Al-Moghrabi and co-workers to release all details of their observations so that formal statistical assessment can be made. Further, it is important to note that we currently lack data from males (newborns as well as fathers), which may also be needed to address the topic of methylation transmission.

Summarizing the evidence, while germline mutations, as reported by Evans and colleagues, may be associated with *BRCA1* constitutive methylation, most likely, this accounts for a small fraction of affected individuals only. As for the rest, we lack conclusive information regarding the cause as well as the risk of potential transmission between parents and children. In this respect, apart from a higher incidence of constitutive methylation in the general population, current findings for *BRCA1* methylation resemble previous findings for *MLH1* methylation and the risk of colorectal cancer [13, 14].
